# Clinical predictors and electrodiagnostic characteristics in patients with Guillain-Barré syndrome with respiratory failure: a retrospective, matched case-control study

**DOI:** 10.7717/peerj.12930

**Published:** 2022-02-10

**Authors:** Kanchana Charoentanyarak, Apiradee Singjam, Jittima Saengsuwan

**Affiliations:** 1 Rehabilitation Medicine Unit, Khon Kaen Hospital, Khon Kaen, Thailand; 2 Department of Rehabilitation Medicine, Faculty of Medicine, Khon Kaen University, Khon Kaen, Thailand

**Keywords:** Association, Case-control, Electrodiagnostic study, Guillain-Barré syndrome, Mechanical ventilator

## Abstract

**Background:**

Respiratory failure is a common complication of Guillain-Barré syndrome (GBS). This study aimed to determine the clinical predictors and electrodiagnostic (EDx) characteristics in patients with Guillain-Barré syndrome (GBS) with respiratory failure.

**Methods:**

The retrospective study included 29 confirmed GBS cases with respiratory failure and age- (±5 years) and sex-matched controls (1:1). The dependent *t*-test and McNemar–Bowker test were used to analyse the continuous and categorical data, respectively. In addition, a multiple logistic regression analysis was used to analyse the predictive factors for respiratory failure.

**Results:**

Among both cases and controls, the majority were male (72.4%), and the average age was 50.9 years. The data showed that patients with respiratory failure had higher GBS disability scores, lower motor power (≤3) of the hip flexors and ankle dorsiflexors, and experienced facial and bulbar palsy. In the multivariate analysis, the significant predictive factors were bulbar palsy (AOR 10.4 [95% CI [2.6–41.4]) and motor power of hip flexors ≤ 3 (AOR 31.4 [95% CI [3.1–314.5]). Patients with respiratory failure had lower compound muscle action potential amplitude of the ulnar and tibial nerves. The median, ulnar, and tibial nerve conduction studies were more likely to reflect inexcitability. The GBS subtypes in GBS patients with and without respiratory failure were not significantly different.

**Conclusions:**

Bulbar palsy and motor power of the hip flexors ≤ 3 were significant predictors for respiratory failure. The GBS subtypes in patients with and without respiratory failure were not significantly different.

## Introduction

Guillain-Barré syndrome (GBS) is an immune-mediated neurological condition characterised by acute or sub-acute progressive symmetric bilateral muscular weakness and areflexia (Hughes & Cornblath, 2005). The worldwide incidence of GBS is approximately 1.1–1.8 cases per 100000 (McGrogan et al., 2009). Incidence increases with age and is higher in men (Sejvar et al., 2011; Kasemsap et al., 2021). Respiratory failure is a common complication of GBS, caused by respiratory muscle weakness and impaired secretion clearance (Green, Baker & Subramaniam, 2018). The prevalence of respiratory failure in GBS ranges from 13.0–50.9% (Malaga et al., 2021; Shangab & Al Kaylani, 2021; Sharshar et al., 2003; Sudulagunta et al., 2015; Toamad et al., 2015; Umer et al., 2019; Ning et al., 2020). According to a recent meta-analysis, the clinical risk factors for respiratory failure in patients with GBS are a short time from symptom onset to hospital admission, bulbar or neck weakness, and severe muscle weakness on admission (Green, Baker & Subramaniam, 2018).

GBS has several subtypes with distinct clinical, pathological, and electrophysiological features, namely: acute inflammatory demyelinating polyradiculoneuropathy (AIDP), acute motor axonal neuropathy (AMAN), acute motor-sensory axonal neuropathy (AMSAN), and Miller Fisher syndrome (Hughes & Cornblath, 2005; McGrogan et al., 2009). An electrodiagnostic (EDx) study is the cornerstone for diagnosing GBS subtypes and helps understand the pathophysiology and assess the prognosis of GBS (Uncini & Kuwabara, 2018). In Western countries, the most common subtype, which accounts for approximately 55–80% of cases, was AIDP (Doets et al., 2018). By comparison, in Asian countries, the proportion of AIDP ranged lower (40–54%), and the axonal type was up to 66.7% (Areeyapinan & Phanthumchinda, 2010; Ye et al., 2010; Verma et al., 2013; Doets et al., 2018).

Studies of EDx characteristics and predictors for GBS with respiratory failure are limited. For example, Durand et al. (2006) found that demyelinating GBS was more common in patients who were mechanically ventilated (MV) (85% *vs.* 51%) in France (Durand et al., 2006). In contrast, a univariate analysis from a prospective study in Bangladesh showed that the presentation of an axonal variant was a significant risk factor for MV (Islam et al., 2019). Since the proportion of GBS subtypes is different in Western and Asian countries, the characteristics of GBS with respiratory failure may be different. Thus, the aim of this study was to 1) determine clinical predictors in patients with GBS with respiratory failure; 2) examine the difference in EDx characteristic in patients with GBS with respiratory failure; and, 3) determine whether there were differences in GBS subtypes among patients with and without respiratory failure.

## Materials & Methods

A retrospective matched case-control study was conducted at Khon Kaen Hospital, Thailand, between March 2020 and March 2021. The study was reviewed and approved by the Khon Kaen Hospital Ethics Committee for Human Research (KEXP63008), performed according to the ethical principles described in the Declaration of Helsinki, and all methods were performed following the relevant guidelines and regulations. The study population included patients who underwent an electrodiagnostic (EDx) study between January 2013 and December 2019. We designed the study to have 1:1 matching. The matching criteria were age in years (± 5 years) and sex. The inclusion criteria were age ≥ 18 years, underwent EDx study, and diagnosed with GBS. The exclusion criteria were pure Miller Fisher syndrome, another diagnosis (*i.e.,* diabetic polyneuropathy, myopathy), or incomplete history record.

The data recorded included date of onset, clinical symptoms, discharge date, and GBS disability score (Hughes et al., 1978) on admission. EDx studies were performed by board-certified rehabilitation physicians using the Nicolet Biomedical Viking quest system (Nicolet Biomedical, Madison, WI, USA). GBS was diagnosed by the criteria proposed by Rajabally et al., which includes the following classifications: acute inflammatory demyelinating polyradiculoneuropathy (AIDP), axonal GBS, equivocal, and normal. Axonal GBS is further subclassified to acute motor axonal neuropathy (AMAN), acute motor-sensory axonal neuropathy (AMSAN), and inexcitable (Rajabally et al., 2015; Uncini & Kuwabara, 2018). A detailed description of the criteria is in the supplementary material. Sural sparing was defined by a greater decrease in the median and or ulnar SNAP compared to the decrease in sural SNAP (Umapathi et al., 2015). Respiratory failure was defined as a need for invasive MV within 30 days after admission (Sharshar et al., 2003).

### Sample size calculation

The sample size calculation was based on a previous study which found that patients with AIDP subtypes comprise 85% of ventilator patients and 51% of non-ventilator patients (34% difference). The study was designed to achieve an 80% power for detecting differences with a 2-sided type I error of 5% (Durand et al., 2006) with a two-sided type I error of 5%. According to these criteria, the estimated sample size was 58 patients (29 per arm).

### Statistical analysis

Continuous data were presented as means and standard deviations. Categorical data were presented as frequencies and percentages. A dependent *t*-test was used to compare the continuous paired data, and the student’s *t*-test was used to compare the continuous data from the sensory SNAP, which cannot be paired because there were multiple instances of no electrical response data. As for nerve conduction parameters, when data was available from both the left and right, the data were randomised to select one side from each individual. McNemar’s test was used to compare the paired data, while the Chi-square test was used for non-matched comparisons of proportions. When the *p*-value was <0.2, these variables were included in the univariate analysis. Multivariate logistic regression models were used to analyse predictive factors for GBS with respiratory failure. Adjusted odds ratios (AOR) with 95% confidence intervals (CI) were calculated, and values of *p* < 0.05 were considered significant. Statistical analyses were performed using Stata version 13.1 (Stata Statistical Software: Release 13. College Station, TX: StataCorp LP.).

## Results

Fifty-eight patients diagnosed with GBS were enrolled, of whom 42 (72.4%) were male. The male-to-female ratio was 2.6:1. The respective mean age was 51.2 and 50.6 in GBS patients with and without respiratory failure. The emergence of cases was more common in the winter and rainy seasons compared to summer. The three most common antecedent events were fever (22.4%), upper respiratory tract infection (15.5%), and diarrhea (13.8%) (Table 1).

**Table 1:** Demographic data of participants (*n* = 58).

**Variables**	**Respiratory failure** **(*n* = 29)**	**No respiratory failure** **(*n* = 29)**	***P* value**
Age (years; mean (SD))	51.2 (15.5)	50.6 (15.1)	Matching criteria
Sex, Male/Female	21 (72.4)/8 (27.6)	21 (72.4)/8 (27.6)	Matching criteria
Smoking	11 (37.9)	12 (41.4)	1.0
Alcoholic drinking	12 (41.4)	14 (48.3)	0.77
Comorbidity			NA
HIV	1 (3.4)	1 (3.4)	1.0
Diabetes mellitus	5 (17.2)	4 (13.8)	1.0
Hypertension	11 (37.9)	4 (13.8)	0.07
Dyslipidemia	3 (10.3)	1 (3.4)	0.61
Autoimmune	1 (3.4)	2 (6.9)	1.0
Hepatitis B	1 (3.4)	3 (10.3)	0.61
Season			0.64
Summer	5 (18.5)	7 (24.1)	
Rainy	13 (48.1)	12 (41.4)	
Winter	9 (31.0)	10 (34.5)	
Antecedent event			
Diarrhea	4 (13.8)	4 (13.8)	1.0
URI symptoms	1 (3.4)	8 (27.6)	0.15
After vaccination	1 (3.4)	0 (0)	1.0
Fever	7 (24.1)	6 (20.7)	1.0

**Notes.**

Data are presented in n (%) unless otherwise specified.

All patients had lower limb weakness (100.0%), and most had numbness (69.0%). Patients with respiratory failure had lower motor power in both the upper and lower extremities and were more likely to have facial or bulbar palsy. The GBS disability score was higher in the respiratory failure group (Table 2). The univariate analysis showed the following were associated with respiratory failure: facial palsy (OR 4.5 [95% CI [1.4–13.7]]), bulbar palsy (OR 7.0 [95% CI [2.2–22.2]]), motor power ≤ 3 in hip flexors (OR 19.8 [95% CI [2.4–165.8]]), and ankle dorsiflexors (OR 3.4 [95% CI [1.0–11.4]]) together with GBS disability scale ≥ 4 (OR 17.1 [95% CI [2.0–144.1]]). In the multivariate analysis, the significant predictive factors were bulbar palsy (AOR 10.4 [95% CI [2.6–41.4]]) and motor power of hip flexors ≤ 3 (AOR 31.4 [95% CI [3.1–314.5]]) (Table 3).

**Table 2:** Clinical signs and symptoms in GBS with and without respiratory failure (*n* = 58).

**Clinical signs and symptoms**	**Respiratory failure** **(*n* = 29)**	**Controls** **(*n* = 29)**	**Mean difference (95% CI)**	***P* value**
Symptom duration before admission (days), mean (SD)	5.5 (4.0)	7.9 (5.1)	−2.4 (−4.8 to 0.0)	0.051
Symptom duration before admission ≤ 7 days	24 (82.8)	20 (69.0)	NA	0.22
Motor power on admission, mean (SD)				
Shoulder abductors	2.8 (1.4)	3.6 (1.0)	−0.9 (−1.6 to 0.1)	0.020
Wrist extensors	2.8 (1.5)	3.8 (1.2)	−1.0 (−1.8 to 0.3)	0.009
Hip flexors	1.7 (1.1)	3.2 (0.8)	−1.5 (−2.0 to 1.0)	<0.001
Ankle dorsiflexors	2.0 (1.3)	3.4 (1.0)	−1.4 (−1.9 to 0.9)	<0.001
Motor power on admission grade ≤ 3				
Shoulder abductors	17 (58.6)	12 (41.4)	NA	0.19
Wrist extensors	17 (58.6)	12 (41.4)	NA	0.19
Hip flexors	28 (96.6)	17 (58.6)	NA	0.001
Ankle dorsiflexors	24 (82.8)	17 (58.6)	NA	0.043
Impair or absent pinprick sensation	19 (65.5)	21 (72.4)	NA	0.26
Facial palsy	17 (58.6)	7 (24.1)	NA	0.013
Bulbar palsy	22 (69.0)	9 (31.0)	NA	0.007
Oculoplegia	5 (17.2)	1 (3.4)	NA	0.13
Autonomic dysfunction	5 (17.2)	2 (6.9)	NA	0.38
Hyporeflexia or areflexia of UE	29 (100)	25 (86.2)	NA	0.41
Hyporeflexia or areflexia of LE	29 (100)	27 (93.1)	NA	0.57
GBS Disability score on admission, mean (SD)	4.4 (0.6)	3.5 (0.7)	0.9 (0.5 to 1.2)	<0.001
GBS Disability score on admission ≥ 3	28 (96.6)	18 (62.1)	NA	0.001
CSF hyperalbuminemia	24 (82.8)	22 (75.9)	NA	0.73

**Notes.**

Abbreviations GBS Guillain-Barré syndrome UE upper extremities LE lower extremities SD standard deviation

Data are presented in n (%) unless otherwise specified.

**Table 3:** Unadjusted and adjusted odds ratio between different variables and respiratory failure in GBS.

	Unadjusted		Adjusted	
	OR (95% CI)	*P* value	OR (95% CI)	*P* value
Duration of symptoms ≤ 7 days	2.2 (0.6–7.5)	0.23		
Shoulder abductors gr ≤ 3	2.0 (0.7–5.7)	0.19		
Wrist extensors gr ≤ 3	2.0 (0.7–5.7)	0.19		
Hip flexors gr ≤ 3	19.8 (2.4–165.8)	0.006	31.4 (3.1–314.5)	0.003
Ankle dorsiflexors gr ≤ 3	3.4 (1.0–11.4)	0.049		
Bulbar palsy	7.0 (2.2–22.2)	0.001	10.4 (2.6–41.4)	0.001
Facial palsy	4.5 (1.4–13.7)	0.009		
GBS disability score on admission ≥ 4	17.1 (2.0–144.1)	0.009		

**Notes.**

Abbreviations GBS Guillain-Barré syndrome OR odds ratio CI confidence interval

An EDx study was done within seven days in a respective 51.7% and 37.9% of patients with and without respiratory failure (*p* = 0.051). Overall, the most common inexcitable motor nerve was the peroneal nerve (51.0%), and the most common inexcitable sensory nerve was the median nerve (66.2%). The median motor, ulnar motor, ulnar sensory, and tibial nerves were more likely to be inexcitable in patients with respiratory failure. The CMAP amplitude of the ulnar and tibial nerves was smaller in patients with respiratory failure. Both groups showed a similar percentage of the sural sparing pattern (25% *vs.* 21.4%) (Table 4). Axonal GBS predominated in both groups (48.3%), and no difference was found in the proportion of axonal and demyelinating GBS (Table 5).

**Table 4:** Electrodiagnostic results in GBS with and without respiratory failure.

**Electrodiagnostic results**	**Respiratory failure** **(*n* = 29)**	**Controls** **(*n* = 29)**	**Mean difference (95% CI)**	***P* value**
Duration from onset to EDx study (days)	7.0 (5.5–13.5)	8.0 (4.0–9.5)	NA	0.079
EDx ≤ 7 days, n (%)	15 (51.7)	11 (37.9)	NA	0.51
**Motor Median NCS (*n* = 15)**				
Latency (ms)	9.4 (5.2)	6.7 (3.5)	2.7 (−1.1 to 6.5)	0.15
NCV (m/s)	44.2 (10.6)	45.3 (13.0)	−1.1 (−8.4 to 6.2)	0.75
CMAP (mV)	2.0 (2.1)	3.3 (3.1)	−1.4 (−3.4 to 0.7)	0.17
No response, n/total n (%)^*^	15/33 (45.5)	5/44 (11.4)	NA	0.001
**Motor Ulnar nerve (*n* = 20)**				
Latency (ms)	5.1 (2.0)	3.7 (1.7)	1.4 (−0.01 to 2.8)	0.052
NCV (m/s)	46.2 (12.9)	54.2 (9.3)	−8.0 (−15.9 to −0.1)	0.048
CMAP (mV)	1.9 (1.6)	3.8 (2.5)	−1.9 (−3.4 to −0.4)	0.015
No response, n/total n (%)^*^	9/33 (27.3)	2/45 (4.4)	NA	0.004
**Motor Peroneal nerve (*n* = 8)**				
Latency (ms)	7.2 (2.0)	6.1 (3.2)	1.0 (−2.1 to 4.1)	0.47
NCV (m/s)	33.5 (15.1)	43.9 (8.3)	−10.4 (−24.7 to 3.8)	0.13
CMAP (mV)	1.2 (0.9)	1.9 (1.2)	−1.8 (−2.0 to 0.5)	0.21
No response, n/total n (%)^*^	30/50 (60.0)	21/50 (42.0)	NA	0.072
**Motor Tibial nerve (*n* = 20)**				
Latency (ms)	7.7 (2.9)	5.0 (2.7)	2.7 (0.8 to 4.5)	0.008
NCV (m/s)	35.4 (12.3)	38.9 (10.1)	−3.5 (−10.7 to 3.7)	0.32
CMAP (mV)	1.5 (1.5)	4.3 (3.4)	−2.7 (−4.6 to −0.9)	0.006
No response, n/total n (%)^*^	13/51 (25.5)	5/50 (10.0)	NA	0.042
**Sensory Median nerve**				
SNAP (µV)^a^ (*n* = 9)	21.5 (13.9) (*n* = 6)	17.3 (7.5) (*n* = 12)	4.2 (−6.3 to 14.7)	0.51
No response, n/total n (%)^*^	21/28 (75.0)	24/40 (60.0)	NA	0.198
**Sensory** **Ulnar nerve**				
SNAP (µV)^a^	17.8 (8.6) (*n* = 6)	14.3 (8.7) (*n* = 19)	3.5 (−4.9 to 11.9)	0.40
No response, n/total n (%)^*^	22/29 (75.9)	15/40 (37.5)	NA	0.002
**Sensory** **Sural nerve**				
SNAP (µV)^a^	18.0 (6.4) (*n* = 10)	12.0 (6.8) (*n* = 12)	6.0 (−0.1 to 11.9)	0.047
No response, n/total n (%)^*^	30/45 (66.7)	26/45 (57.8)	NA	0.38
Sural sparing, n/total n (%)	7/28 (25%)	6/28 (21.4%)	NA	1.00
**Needle EMG study**				
Axonal denervation, n/total n (%)^*^	9/25 (36.0)	6/24 (25.0)	NA	0.40

**Notes.**

Abbreviations CMAP compound muscle action potential NCV nerve conduction velocity EMG electromyography NA not applicable SNAP sensory nerve action potential

The data were analysed by dependent *t*-test unless otherwise specified.

* Chi-square test.

a Student’s *t*-test.

Data are presented in mean (SD) unless otherwise specified.

**Table 5:** GBS classification in GBS with and without respiratory failure.

**GBS subtypes**	**Respiratory failure** **(*n* = 29)**	**Controls** **(*n* = 29)**	***P* value**
AIDP	10 (34.5)	10 (34.5)	0.67
Axonal GBS	14 (48.3)	14 (48.3)	
AMAN	7 (24.1)	6 (20.7)	
AMSAN	7 (24.1)	7 (24.1)	
Inexcitable	0 (0.0)	1 (3.4)	
Equivocal	4 (13.8)	1 (3.4)	
Normal	1 (3.4)	4 (13.8)	

**Notes.**

Abbreviations GBS Guillain-Barré syndrome AIDP Acute inflammatory demyelinating polyradiculoneuropathy, AMAN acute motor axonal neuropathy, AMSAN acute motor sensory axonal neuropathy

Data are presented in n (%).

## Discussion

Compared to previous epidemiological studies in Thailand, our patients with respiratory failure were older (50.6 *vs.* 42.0–43.0 years), which correlates to previous findings in which the mortality rate in the older age group was higher than the younger age group (5.2% in patients age ≥ 65 years compared to 1.5% and 3.6% in patients age ≥ 18 and 19–64 years, respectively) (Areeyapinan & Phanthumchinda, 2010; Kasemsap et al., 2021; Wen et al., 2021). Similarly, Shangab & Al Kaylani (2021) reported that older age at presentation is a major predictor for the need for mechanical ventilation (MV). Additionally, we found a higher male-to-female ratio of 2.6:1 compared to 1.6:1 in overall GBS, suggesting that males might have a higher morbidity than females (Kasemsap et al., 2021). This observation is different from a meta-analysis that revealed that men were no more likely to require MV (Green, Baker & Subramaniam, 2018) but was consistent with previous studies in Bangladesh and the United Arab Emirates, which showed that 68–78% of ventilated patients were male (Shangab & Kaylani, 2021; Islam et al., 2019). The seasonal prevalence of GBS was comparable to previous studies confirming that the rainy and winter seasons had significantly more patients (Areeyapinan & Phanthumchinda, 2010; Kasemsap et al., 2021).

The following factors are related to respiratory failure: rapid disease progression, weakness of respiratory muscles, lower Medical Research Council (MRC) score at nadir, a short time from symptom onset to hospital admission, facial palsy, neck weakness, bilateral facial weakness, autonomic dysfunction, and bulbar palsy (Lawn et al., 2001; Durand et al., 2006; Green, Baker & Subramaniam, 2018; Wen et al., 2021). Our univariate analysis revealed significant factors: weakness in the hip flexors and ankle dorsiflexors, higher GBS disability score on admission, bulbar palsy and facial palsy. However, the multivariate analysis showed that the only significant predictors were bulbar palsy (AOR 10.4) and weakness of hip flexors of MRC ≤ 3 (AOR 31.4). By comparison, bulbar weakness was a significant predictor of respiratory failure in several studies (Malaga et al., 2021; Kanikannan et al., 2014; Toamad et al., 2015; Wu et al., 2015; Green, Baker & Subramaniam, 2018; Islam et al., 2019; Umer et al., 2019; Luo et al., 2020; Ning et al., 2020). Although the short time from symptom onset to admission (≤7 days) was a significant predictor in several studies (Rantala et al., 1995; Toamad et al., 2015; Wu et al., 2015; Green, Baker & Subramaniam, 2018; Umer et al., 2019; Luo et al., 2020), it did not reach statistical significance in our univariate and multivariate analyses. This may be because our hospital is a tertiary hospital, and some patients were referred from community hospitals, so our hospital admission date may not be the date of initial hospital admission. Our study showed that hip flexors weakness ≤ 3 was a strong predictor for respiratory failure, which agrees with Walgaard et al. (2010) and Wu et al. (2015) who showed that lower muscle power was a risk factor for MV. In a recent meta-analysis, increased risk of intubation was associated with a short time from symptom onset to hospital admission, bulbar involvement or neck weakness, and severe muscle weakness at hospital admission. Facial weakness and autonomic dysfunction were not significant predictors after multivariable analysis (Green, Baker & Subramaniam, 2018).

The findings of lower CMAP amplitude and inexcitable motor nerve conduction agree with Sundar et al. (2005) who found markedly attenuated compound muscle action potentials and inexcitable motor nerves were more common in the ventilated group, and Walgaard et al. (2010) who showed that patients with unexcitable nerves on nerve conduction study had a greater chance of requiring prolonged MV. However, we did not investigate the role of EDx features as predictive factors because the results of the matched data were not complete leading to a small sample size among groups.

A sural sparing pattern was found in 25% of mechanically ventilated patients and 21.4% in those who were not. Our results agree with those of Rasera et al. (2021) who found a sural sparing pattern in 21% of patients with GBS in Italy, somewhat higher than the respective 15% and 16.7%% reported by Sharma et al. (2016) in India and Gómez-Piña et al. (2021) in Mexico (16.7%). Notwithstanding, these findings are lower than previous studies where the sural sparing pattern in GBS patients ranged between 34.4 and 72% (Al-Shekhlee, Robinson & Katirji, 2007; Derksen et al., 2014; Ahdab et al., 2018). The distribution of GBS subtypes may play a role, as we found a higher occurrence of axonal subtypes than other studies where the sural sparing pattern was more common in AIDP (Yadegari, Nafissi & Kazemi, 2014; Sharma et al., 2016; Gómez-Piña et al., 2021; Mani et al., 2021). In addition, the difference in findings may be related to the timing of Edx since previous studies demonstrated an increase in the sural sparing pattern with serial EDx (Gupta et al., 2008; Umapathi et al., 2015).

Regarding EDx results, we found no difference in GBS subtypes in the group with respiratory failure, which is consistent with some studies (Green, Baker & Subramaniam, 2018; Parveen et al., 2020) but contradicts others where a higher proportion of demyelinating (Durand et al., 2006; Yamagishi et al., 2017) or axonal GBS subtypes was found (Shangab & Al Kaylani, 2021; Walgaard et al., 2010; Islam et al., 2019; Luo et al., 2020).

We examined the predictive factors for respiratory failure in patients with GBS using a case-control study design, which is useful when investigating uncommon diseases. Since age and sex were matching criteria, we could not establish the role of these variables as potential predictors, although we found that older age and men seemed to more commonly have respiratory failure. Another limitation of case-control studies is the inability to show the temporal relationship between factors and outcomes because cases and controls are investigated after the diagnosis. Since our study employed previously recorded data, some missed factors might have been potential risk/predictive factors associated with respiratory failure in GBS (*i.e.,* neck flexor weakness) (Umer et al., 2019) or parameters of pulmonary function (*i.e.,* vital capacity) (Sharshar et al., 2003; Durand et al., 2006; Kanikannan et al., 2014). Additionally, although a serial EDx study was not done, if it were, it might lead to a change in the EDx due to a resolution of reversible conduction failure or misclassification of subtypes (Uncini et al., 2017; Leonhard et al., 2019; Mani et al., 2021). The generalizability of data may be limited because it was a single-centre source. Moreover, we had small samples for each EDx parameter limiting the ability to integrate EDx results in the multivariate analysis. The main limitation was the lack of a temporal relationship between the clinical/electrodiagnostic features of GBS and respiratory failure, pointing out the need for a well-designed prospective study.

## Conclusion

Bulbar palsy and motor power of the hip flexors ≤ 3 were significant predictors for respiratory failure. No significant difference in GBS subtypes was found in patients with and without respiratory failure.

## Supplemental Information

10.7717/peerj.12930/supp-1 Supplemental Information 1 Clinical and electrodiagnostic characteristics in patients with GBS with respiratory failure

10.7717/peerj.12930/supp-2 Supplemental Information 2 Electrodiagnostic criteria for GBS

## Additional Information and Declarations

### Data Availability

The following information was supplied regarding data availability: The raw data are available in the Supplemental File.
