# Supplementary material for: Clinical predictors and electrodiagnostic characteristics in patients with Guillain-Barré syndrome with respiratory failure: a retrospective, matched case-control study
Source: PeerJ. 2022 Feb 10;10:e12930. doi: 10.7717/peerj.12930 (PMC8841033; doi:10.7717/peerj.12930)
Supplement: Supplemental Information 2 [file peerj-10-12930-s002.pdf]

Supplementary material

Electrodiagnostic criteria for GBS (Rajabally et al., 2015)

1. Normal (All the following in all nerves tested)

- DML≤100% ULN
- F-wave present with latency≤100% ULN
- MCV≥100% LLN
- Distal CMAP≥100% LLN
- Proximal CMAP/distal CMAP ratio >0.7 (excluding the tibial nerve)

2. Acute inflammatory demyelinating polyradiculoneuropathy (AIDP)

- At least one of the following in at least two nerves:

– MCV <70% LLN

– DML >150% ULN

– F-response latency >120% ULN, or >150% ULN (if distal CMAP <50% of LLN)

- OR

– F-wave absence in two nerves with distal CMAP ≥20% LLN, with an additional parameter, in one other nerve.

- OR

– Proximal CMAP/distal CMAP ratio <0.7 (excluding the tibial nerve), in two nerves with an additional parameter, in one other nerve

3. Axonal GBS forms (Uncini and Kuwabara 2018)

3.1 AMAN GBS:

- None of the above features of demyelination in any nerve (except one demyelinating feature allowed in one nerve if distal CMAP <10% LLN), and at least one of the following:

– Distal CMAP <80% LLN in two nerves

– F-wave absence in two nerves with distal CMAP≥20% LLN, in absence of any demyelinating feature in any nerve

– Proximal CMAP/distal CMAP ratio <0.7, in two nerves (excluding the tibial nerve)

– F-wave absence in one nerve with distal CMAP≥20% LLN OR proximal CMAP/distal CMAP ratio <0.7 (excluding the tibial nerve), in one nerve; with INADDITION, distal CMAP <80% LLN in one other nerve

3.2 AMSAN GBS (Rajabally et al. 2015)

The same of AMAN Criteria in motor nerves **plus** SNAP <50% LLN in at least two nerves.

3.3 Inexcitable:

If distal CMAP absent in all nerves (or present in only one nerve with distal CMAP <10% LLN)

4. Equivocal

- Abnormal range findings however not fitting criteria for any other group

(CMAP: compound muscle action potentials; DML: distal motor latency; GBS: Guillain–Barré syndrome; LLN: lower limit of normal; MCV: motor conduction velocity; ULN: upper limit of normal; SNAP: sensory nerve action potential.)

Abbreviations: CMAP, compound muscle action potentials; DML, distal motor latency; GBS, Guillain–Barré syndrome; LLN, lower limit of normal; MCV, motor conduction velocity; ULN, upper limit of normal.

Reference

Rajabally, Y. A., M. C. Durand, J. Mitchell, D. Orlikowski & G. Nicolas (2015) Electrophysiological diagnosis of Guillain-Barré syndrome subtype: could a single study suffice? *J Neurol Neurosurg Psychiatry,* 86**,** 115-9.

Uncini, A. & S. Kuwabara (2018) The electrodiagnosis of Guillain-Barre syndrome subtypes: Where do we stand? *Clinical Neurophysiology,* 129**,** 2586-2593.
